# Pseudocospeciation of the mycoparasite *Cosmospora* with their fungal hosts

**DOI:** 10.1002/ece3.1967

**Published:** 2016-02-09

**Authors:** Cesar S. Herrera, Yuuri Hirooka, Priscila Chaverri

**Affiliations:** ^1^ Department of Plant Science and Landscape ArchitectureUniversity of Maryland2112 Plant Sciences BuildingCollege ParkMaryland20742United States; ^2^ Department of Clinical Plant Science, Faculty of BioscienceHosei University3‐7‐2 Kajino‐choKoganeiTokyoJapan; ^3^ Escuela de BiologíaUniversidad de Costa RicaApartado 11501‐2060San PedroSan JoséCosta Rica

**Keywords:** Ascomycota, coevolution, fungal host, fungal parasite, *Nectria*, Nectriaceae

## Abstract

Species of *Cosmospora* are parasites of other fungi (mycoparasites), including species belonging to the Xylariales. Based on prior taxonomic work, these fungi were determined to be highly host specific. We suspected that the association of *Cosmospora* and their hosts could not be a result of random chance, and tested the cospeciation of *Cosmospora* and the their hosts with contemporary methods (e.g., ParaFit, PACo, and Jane). The cophylogeny of *Cosmospora* and their hosts was found to be congruent, but only host‐parasite links in more recent evolutionary lineages of the host were determined as coevolutionary. Reconciliation reconstructions determined at least five host‐switch events early in the evolution of *Cosmospora*. Additionally, the rates of evolution between *Cosmospora* and their hosts were unequal. This pattern is more likely to be explained by pseudocospeciation (i.e., host switches followed by cospeciation), which also produces congruent cophylogenies.

## Introduction

Evolutionary relationships of fungus–fungus systems have been rarely studied. Millanes et al. (2014) studied the *Biatoropsis* Räsänen‐*Usnea* Dill. ex Adans. system (a fungal parasite–fungal host association) and demonstrated that host‐switch events played a more prevalent role than cospeciation events in their reconciliation reconstructions of *Biatoropsis* and *Usnea* phylogenies. In addition, the fungal cultivars of the fungus‐growing ants (fungi belonging to the Agaricaceae and Tricholomataceae) and their fungal parasites, *Escovopsis* J.J. Muchovej and Della Lucia, have highly congruent phylogenies (Currie et al. 2003). A few other examples of fungus–fungus systems include *Eudarluca‐Ampelomyces* (Nischwitz et al. 2005), *Squamanita*‐*Cystoderma* (Matheny and Griffith 2010), and *Xerocomus‐Hypomyces* (Douhan and Rizzo 2003). In other nonfungal systems, host–parasite relationships have also produced congruent cophylogenies (e.g., Clayton and Johnson 2003; Banks et al. 2006; Hosokawa et al. 2006; Hughes et al. 2007; Marussich and Machado 2007; Noda et al. 2007; Jackson et al. 2008; Lanterbecq et al. 2010; Göker et al. 2011), which have been taken as evidence of cospeciation between hosts and parasites. However, congruent cophylogenies can also result from other evolutionary mechanisms besides cospeciation such as coevolution and sequential evolution. Coevolution is the evolution in two or more species that leads to reciprocal evolutionary changes, and in sequential evolution, changes in one taxon lead to changes in the other taxon, but the change is not reciprocal (reviewed in Ridley 2007). Cospeciation involves the joint speciation of two or more species that are ecologically associated (e.g., host–parasites; Page 2003). There are also evolutionary events that would lead to incongruent cophylogenies: (1) duplication (independent speciation), (2) host switching, and (3) lineage sorting (e.g., extinction and “missing the boat”; reviewed in Page 2003 and Paterson and Banks 2001).

**Figure 1:**
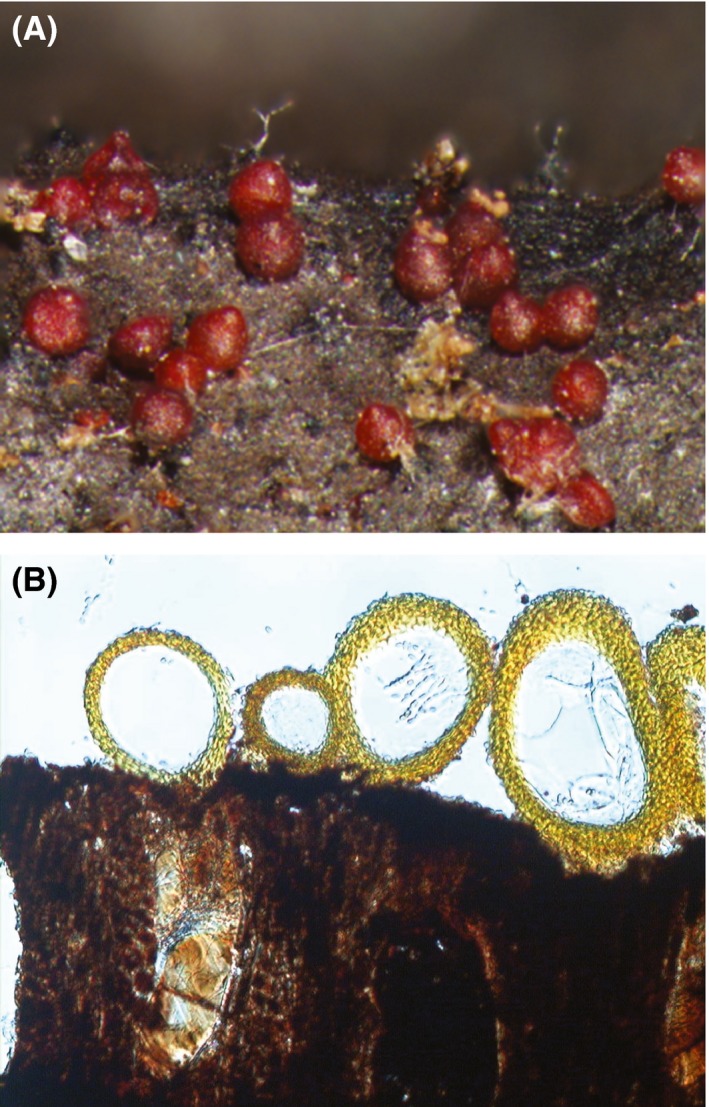
*Cosmospora* species. (A) *Cosmospora* perithecia (reddish). (B) Median section of *Cosmospora* perithecia (stained in Lactic Acid: yellow) and fruiting body of xylariaceous host (dark). *Cosmospora* perithecia growing directly above the host perithecia (empty spaces).

In the present study, we studied the association between species of *Cosmospora* Rabenh. (sensu lato; Ascomycota, Hypocreales, Nectriaceae; a mycoparasite, which means a fungus that parasitizes other fungi) and their fungal hosts. *Cosmospora* is a fungal genus that at some point was determined to be polyphyletic and thus segregated into many monophyletic genera (e.g., *Cosmospora* sensu stricto, *Dialonectria*,* Microcera*, and *Pseudocosmospora*, among others; Schoch et al. 2000; Luo and Zhuang 2010; Luo and Zhuang 2012; Gräfenhan et al. 2011; Herrera et al. 2013). The sexual fruiting bodies (perithecia) in *Cosmospora* sensu lato (hereafter “*Cosmospora*‐like”) are highly conserved to the degree of being indistinguishable. Briefly, the perithecia are reddish, small in size (<300 *μ*m), and pear shape (Fig. 1A). The sexual spores (ascospores) are ellipsoid to ellipsoid‐fusiform, one‐septate, yellow‐brown and warted at maturity (Samuels et al. 1991; Rossman et al. 1999). The perithecia usually grow in clusters on other fungi, scale insects, and rarely on wood and herbaceous substrata (Rossman 1983; Samuels et al. 1991; Rossman et al. 1999). *Cosmospora*‐like fungi are reported to be most common in recently disturbed forest stands (Chaverri and Vílchez 2006), and to have much greater diversity in warm temperate and tropical regions (Rossman et al. 1999). However, they are not infrequent outside those regions.

Most *Cosmospora*‐like fungi are mycoparasites of fungi in the families Xylariaceae and Diatrypaceae (Xylariales, Ascomycota; Gräfenhan et al. 2011). Tsuneda (1982) first described the attack by these mycoparasites. Briefly, the fruiting bodies of the fungal host are penetrated by the *Cosmospora* species, and the fleshy insides of the fungal host are slowly attacked and consumed by the *Cosmospora*'s vegetative hyphae. It is thought that the slow attack ensures an extended period of nutrient uptake. The fungal host is able to mature but not to release ascospores. Ultimately, the host's fleshy insides are replaced by vegetative hyphae of the *Cosmospora*. The mycoparasitic attack ends with the formation of its own perithecia directly on the surface of the host's fruiting bodies (Fig. 1B), while simultaneously consuming its own vegetative hyphae for the production of perithecia (Tsuneda 1982).

*Cosmospora* sensu stricto include species that grow on xylariaceous fungi (Xylariaceae, Xylariales, Ascomycota). During the taxonomic revision of these fungi (Herrera et al. 2015), it was observed that these species have a high degree of host specificity. Host specificity is broadly defined as an association that does not appear random and where a parasite is capable of infecting one or a few specific hosts. Given this host‐specific trait, we hypothesized that species of *Cosmospora* have cospeciated with their xylariaceous fungal hosts following Fahrenholz’ rule (i.e., host and parasites form cophylogenies; reviewed in Ridley 2007). We investigated this hypothesis using multilocus phylogenies and reconciliation reconstructions (e.g., ParaFit, PACo, and Jane).

## Methods

### Cosmospora *phylogeny*

Thirteen species were selected based on the availability of host data. Sequences were generated in prior taxonomic work (Herrera et al. 2013, 2015). Briefly, DNA was extracted from mycelium grown for 1 week in Difco^™^ potato dextrose broth with PowerPlant^®^ DNA Isolation Kit (MO BIO Laboratories Inc., Solana Beach, CA). Internal transcribed spacer (ITS), large subunit nuclear ribosomal DNA (LSU), DNA replication licensing factor (*mcm7*), RNA polymerase II subunit one (*rpb1*), and *β*‐tubulin (*tub2*) were amplified in an Eppendorf Mastercycler thermocycler (Eppendorf, Westbury, NY) and sequenced at the DNA Sequencing Facility (Center for Agricultural Biotechnology, University of Maryland, College Park, Maryland). The selected species and the associated sequences are listed in Table S1.

Sequences were aligned via the MAFFT v.6 web service (http://mafft.cbrc.jp/alignment/server/; Katoh et al. 2002; Katoh and Standley 2013) implementing the E‐INS‐i alignment strategy and the 1PAM/*κ *=* *2 scoring matrix for nucleotide sequences. Alignments were manually edited in Mesquite 2.75 (Maddison and Maddison 2011). Ambiguously aligned regions were excluded. The best‐fit partitioning scheme among the sequenced loci and the model of nucleotide substitution for each partition were determined with PartitionFinder v1.1.1 (Lanfear et al. 2012) using the default settings.

Phylogenetic analysis was performed using GARLI v2.01 (Genetic Algorithm for Rapid Likelihood Inference; Zwickl 2006) via the GARLI web service (http://www.molecularevolution.org; Bazinet and Cummings 2011), which uses a grid computing system associated with The Lattice Project (Cummings and Huskamp 2005; Bazinet and Cummings 2008). Fifty independent search replicates were performed to find the best tree with a fast ML stepwise‐addition algorithm. One thousand bootstrap replicates were used in the bootstrap analysis.

### Host phylogeny

Effort was made to extract DNA directly from the fruiting bodies of the host. However, in most cases, we obtained sequences of the associated *Cosmospora* species suggesting that the mycoparasite had already attacked the host. We were able to obtain ITS sequences from the hosts of *Cosmospora khandalensis* and *Pseudocosmospora joca* by amplifying DNA with Illustra GenomiPhi V2 DNA Amplification Kit (GE Healthcare Bio‐Sciences Corp., Piscataway, New Jersey) following the manufacturer's instructions. The identification of the host for *P. joca* was determined to be *Biscogniauxia capnodes* (Xylariaceae), while the host for *C. khandalensis* could only be identified to genus rank as *Annulohypoxylon* (Xylariaceae). In our previous taxonomic revision of the *Cosmospora viliuscula* species complex (Herrera et al. 2015), we conservatively identified the hosts in the complex based on morphological characters. We extracted sequences (ITS, *act*A, *rpb*2, and tub2) from GenBank for these species, and these are listed in Table S2. Phylogenetic analysis was performed as described for the mycoparasite.

### Cophylogenetic analyses and comparison of molecular substitution rates

The presence of cophylogenies or mirror phylogenies was analyzed with various statistical tests by reconstructing phylogenies of selected *Cosmospora* spp. and their corresponding xylariaceous hosts. If cospeciation tests suggest identical speciation times, agreeing topologies, and corresponding relative substitution rates, it could be inferred that coevolution or cospeciation occurred (Huelsenbeck et al. 1997; Legendre et al. 2002; Schardl et al. 2008). An alternative hypothesis would rely on sequential evolution. In sequential evolution, changes in one taxon lead to changes in the other taxon, but not reverse. If sequential evolution occurred, the speciation times and substitution rates would be different in the phylogenies of the host and symbiont, likely resulting in imperfect matches between the phylogeny of the host and symbionts.

Thirteen host species and 13 *Cosmospora* species were included in the cophylogenetic analyses. We performed two distance‐based methods: PACo (Balbuena Díaz‐Pinés et al. 2013) and ParaFit (Legendre et al. 2002). Additionally, two tree‐reconciliation methods were performed: Jane v.4 (Conow et al. 2010) and CoRe‐PA v0.5.1 (Merkle et al. 2010). A tanglegram between *Cosmospora* species and their host was generated with TreeMap v3.0*β* (Charleston 2011).

Distance‐based methods were implemented in R (R Core Team 2013) with the APE package (Paradis et al. 2004). Host and parasite phylogenies were transformed into matrices of patristic distances, and transformed again into principle coordinates to describe the phylogenies. The host principle coordinates, parasite principle coordinates, and host–parasite association matrices were used to test the degree of congruence between the host and parasite phylogenies with a global host–parasite statistic, and the significance of the statistic was determined using a permutation test. 100,000 permutations were run for PACo, whereas 999 permutations were run for ParaFit. PACo and ParaFit algorithms test the null hypothesis that the host and parasite phylogenies are independent (or randomly associated).

In an evaluation of tree‐reconciliation methods (CoRe‐PA, Jane, and TreeMap), CoRe‐PA was determined the most precise tool available in predicting the associations between hosts and parasites, although it does not produce an optimal estimate of the number of cospeciation and switching events. Jane, on the other hand, yielded the correct estimate of cospeciation events (Keller‐Schmidt et al. 2011). Because they are based on the optimality criterion of maximum parsimony, these methods seek to find the cophylogeny with the minimum cost. CoRe‐PA and Jane assign costs to four evolutionary events: cospeciation, duplication, host switch, and sorting. Additionally, Jane assigns a cost to failure to diverge. We used the default cost settings in CoRe‐PA and Jane.

*Tub2* represented the only protein‐coding locus out of two homologous loci available to compare molecular substitution rates. *Cosmospora* and host *tub2* matrices were reduced to 452 and 415 base pairs, respectively. These base pairs represented the homologous region in both *tub2* matrices. Comparison of molecular rates requires that data conform to neutral expectations and to a molecular clock (Hafner et al. 1994). Mega v5.2.2 (Tamura et al. 2011) was used to identify fourfold‐degenerate sites, which are presumed to be neutral, in *Cosmospora* and host matrices, and was used to perform likelihood ratio tests assessing molecular clock with fourfold‐degenerate sites.

Branch lengths were estimated with ML search in RAxML v.8 (Stamatakis 2014) constraining taxa to fit the best *Cosmospora* and host phylogenies using matrices with fourfold‐degenerate sites. Page (1996) argued that branch comparison required homologous events. Branch length comparisons were restricted to host–parasite links that were determined to be coevolutionary in PACo. Branch lengths were compared between cospeciating *Cosmospora* and hosts using Model II regression analysis in R with the package lmodel2 (Legendre 2014). Model II regression analysis determined whether one associate evolved faster or slower than the other, which is assessed by the slope of the relationship, and determined whether *Cosmospora* speciated before, or after its host (assessed by the *y*‐intercept of the relationship; Hafner and Nadler 1990).

## Results

### Phylogenetic analyses

**Figure 2:**
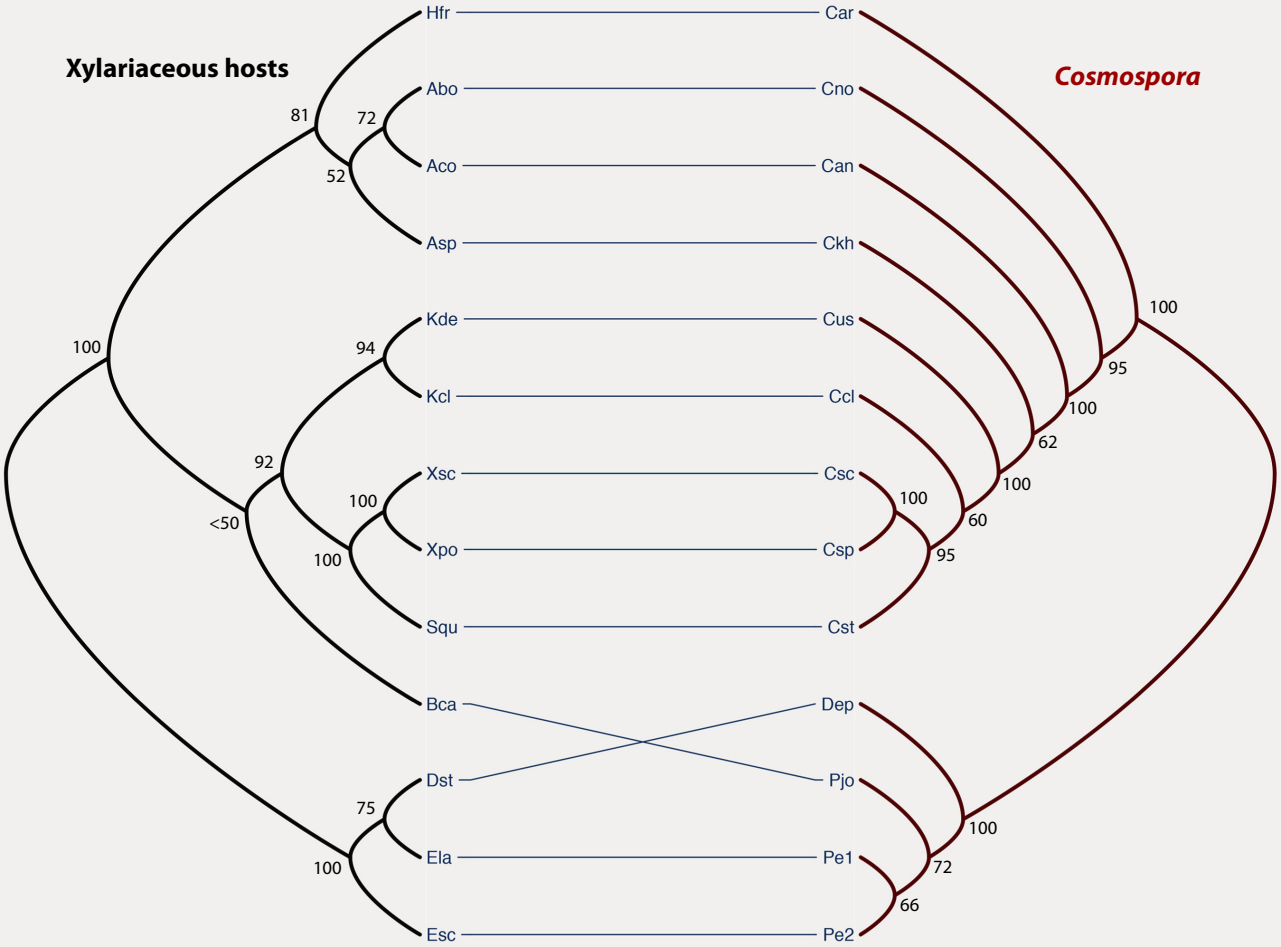
Tanglegram between *Cosmospora* (red, right) and host (black, left) phylogenies. Solid lines between *Cosmospora* species and the host indicate host–parasite associations. ML bootstraps are provided for each node. Taxa abbreviations for hosts are as follows: *Annulohypoxylon bovei* (Abo), *A. cohaerens* (Aco), *Biscogniauxia capnodes* (Bca), *Diatrype stigma* (Dst), *Eutypa lata* (Ela), *Eutypella scoparia* (Esc), *Hypoxylon fragiforme* (Hfr), *H. “khandalensis”* (Asp), *Kretzschmaria clavus* (Kcl), *K. deusta* (Kde), *Stilbohypoxylon quisquiliarum* (Squ), *Xylaria polymorpha* (Xpo), *X. scruposa* (Xsc); for the mycoparasites: *Cosmospora annulohypoxili* (Can), *C. arxii* (Car), *C. clavi* (Ccl), *C. khandalensis* (Ckh), *C. novaezelandica* (Cno), *C. scruposae* (Csc), *Cosmospora* sp. (Csp), *C. stilbohypoxili* (Cst), *C. ustulinae* (Cus), *Dialonectria episphaeria* (Dep), *Pseudocosmospora eutypae* (Pe1), *P. eutypellae* (Pe2), and *P. joca* (Pjo).

PartitionFinder determined three partitions in the *Cosmospora* supermatrix, which included 3217 total characters (ITS: 570; LSU: 782; *mcm7*: 615; *rpb1*: 690; and *tub2*: 560). These partitions were ITS, LSU, and *mcm7 *+* rpb1 *+* tub2*. The best model of nucleotide substitution was TIMef + I, K80 + I, and TrNef + G, for each partition, respectively. The negative log likelihood for the best tree was −10,885.2025. *Cosmospora* lineages were well supported with only three exceptions (Fig. 2).

Three partitions were determined for the host supermatrix that comprised 3159 total characters (ITS: 466; *act*A: 301; *rpb2*: 1199; and *tub2*: 1193). These partitions were ITS, *act*A + *rpb*2, and *tub2*. TIMef + G, K80 + I, and TrN + G were selected as the best models for each partition, respectively. The negative log likelihood for the best tree was −12,636.6736. Lineages of xylariaceous fungi were well supported with only two exceptions (Fig. 2).

### Distance‐based analyses

**Figure 3:**
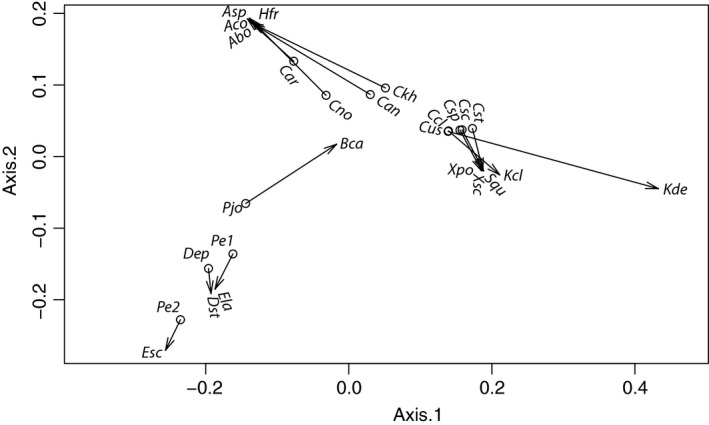
Procrustean superimposition plot of *Cosmospora* and fungal hosts. The ordinations of *Cosmospora* and their fungal hosts are Principal Correspondence Coordinates of patristic distances. The *Cosmospora* configuration (dots) has been rotated and scaled to fit the fungal hosts ordination (arrow tips). See Fig. 2 legend for abbreviations of taxa.

A procrustean superimposition plot of axes one and two, corresponding to patristic distances of *Cosmospora* and their fungal hosts, suggested three groups of host–parasite associations (Fig. 3). One group is composed of *Cosmospora* species associated with *Annulohypoxylon* and *Hypoxylon*. Another group is composed of *Cosmospora* species associated with *Kretzschmaria*,* Stilbohypoxylon,* and *Xylaria*. A third group is composed of *Dialonectria episphaeria*,* Pseudocosmospora,* and their hosts.

**Figure 4:**
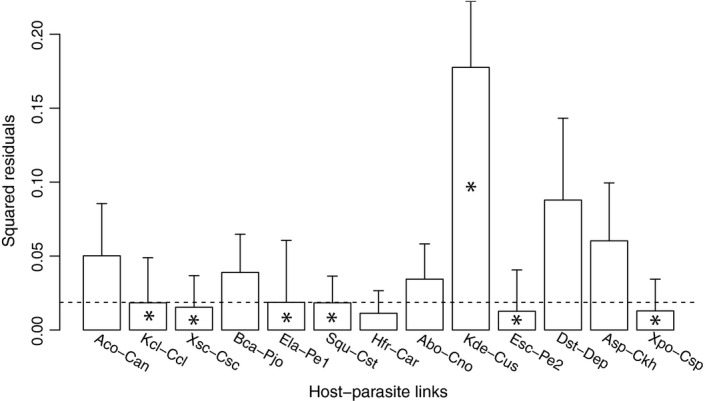
Contributions of individual host–parasite links to the Procrustean fit. Jacknifed squared residuals (bars) and upper 95% confidence intervals (error bars) resulting from applying PACo to patristic distances. Asterisks identify links significantly supported (*α *= 0.05) by ParaFitLink1. The median squared residual value is shown as a dashed line. See Fig. 2 legend for abbreviations of taxa.

Distance‐based methods supported an overall congruence between the phylogenies of *Cosmospora* and their hosts. The PACo analysis produced a residual sum of squares (*m* ^2^_XY_) of 0.4193 with an associated permutational *P *=* *0.00001. Similarly, the ParaFit global fit statistic was 0.0275 (*P *=* *0.005). The contribution of each host–parasite to the global fit was assessed with a jackknife procedure applied in PACo, which estimated the squared residual and its 95% confidence interval of each individual link (Fig. 4). Most links associated with *Kretzschmaria*,* Stilbohypoxylon,* and *Xylaria* hosts contributed relatively little to the residual sum of squares. The *Eutypa lata*‐*Pseudocosmospora eutypae* and *Eutypella scoparia*‐*Pseudocosmospora eutypellae* links were also determined to contribute relatively little to the residual sum of squares. ParaFitLink1 analysis also considered these links + *Kretzschmaria deusta*‐*Cosmospora ustulinae* as coevolutionary at 0.05 significance level.

### Tree‐reconciliation analyses

**Figure 5:**
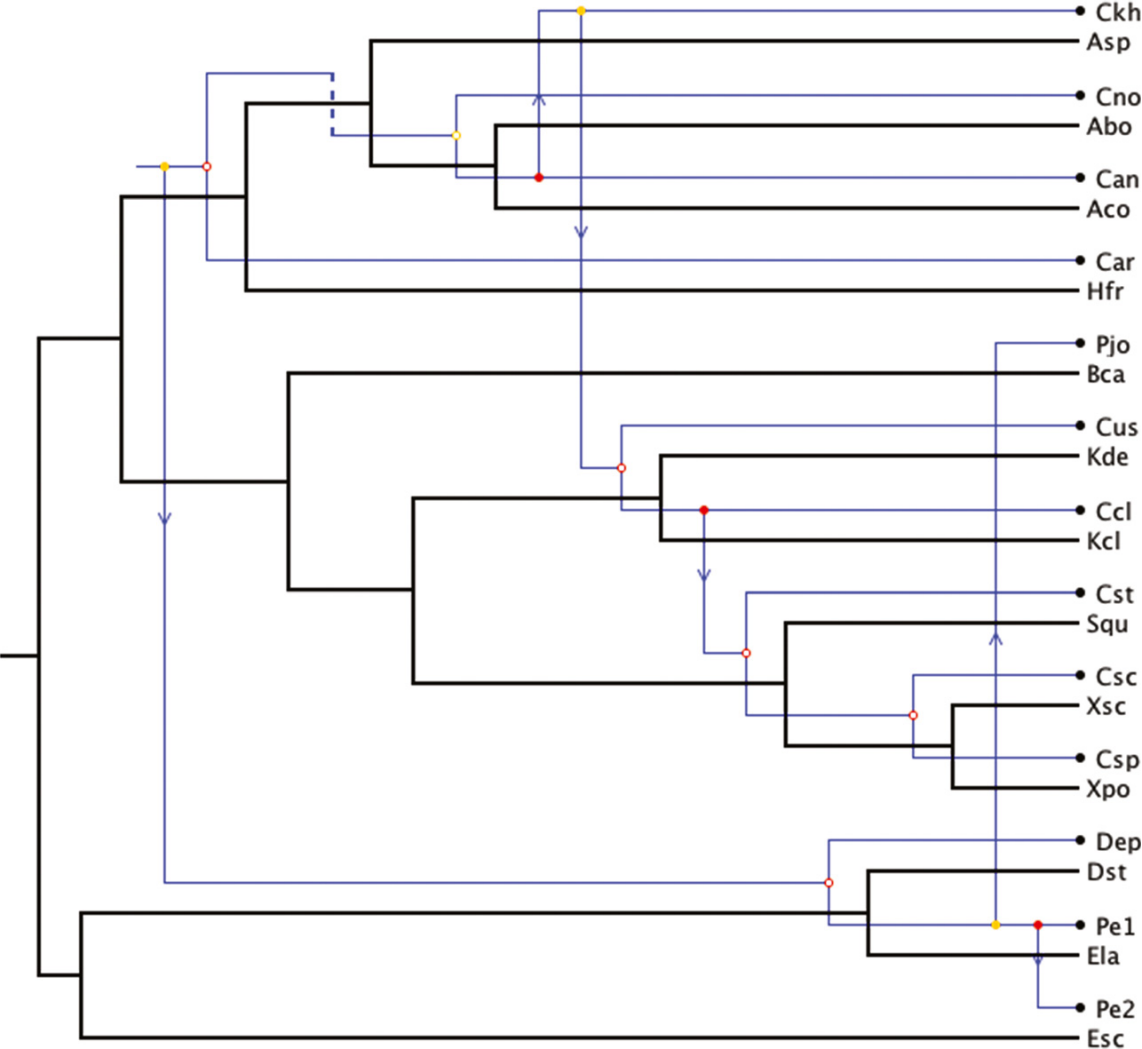
Reconciliation between *Cosmospora* and host phylogenies. One of 263 isomorphic solutions with seven cospeciations, five duplications, and host switches, and three losses (total cost* *=* *13). The reconciliation of *Cosmospora* and host trees was generated with Jane v.4. Blue and black lines represent *Cosmospora* and their fungal hosts, respectively. Empty circles represent cospeciations; arrows represent host switches; and dashed lines represent sorting events. See Fig. 2 legend for abbreviations of taxa.

**Figure 6:**
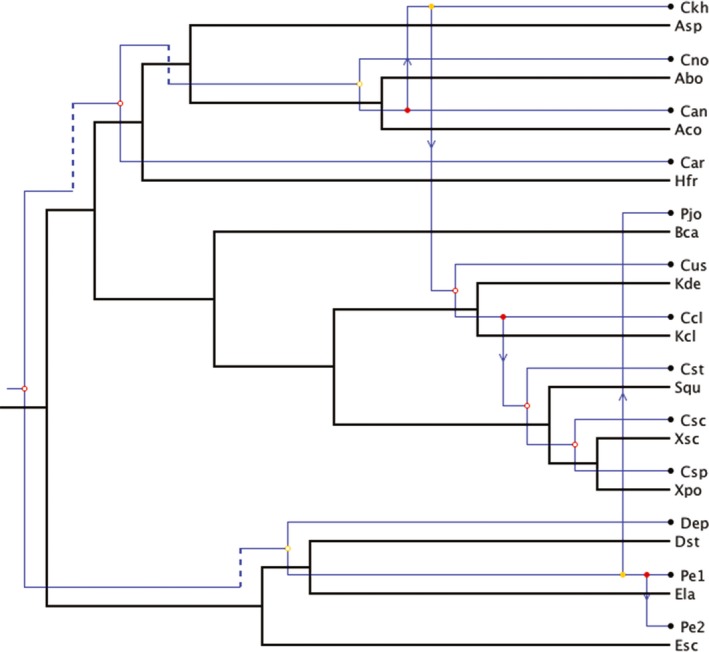
Equally parsimonious reconciliation between *Cosmospora* and host phylogenies. One of 68 isomorphic solutions with six cospeciations, six duplications, and host switches, and one loss (total cost* *=* *13). The reconciliation of *Cosmospora* and host trees was generated with Jane v.4. Blue and black lines represent *Cosmospora* and their fungal hosts, respectively. Empty circles represent cospeciations; arrows represent host switches; and dashed lines represent sorting events. See Fig. 2 legend for abbreviations of taxa.

The Tanglegram between *Cosmospora* and host phylogenies showed some internal congruence (Fig. 2). The reconciliation of the *Cosmospora* tree with the host tree revealed that a maximum of seven cospeciation events might have occurred in their evolution (Fig. 5). This reconciliation also contained five host switches and three sorting events. The total cost for this reconciliation was 18 in CoRe‐Pa and 13 in Jane. Jane generated another equally parsimonious reconciliation between *Cosmospora* and host trees (Fig. 6). This reconciliation had six cospeciations, six host switches, and one sorting event. In only one instance out of 100 did a better random sample solution produced a reconciliation cost below 13 (*P *=* *0.01).

### Comparison of molecular rates

**Figure 7:**
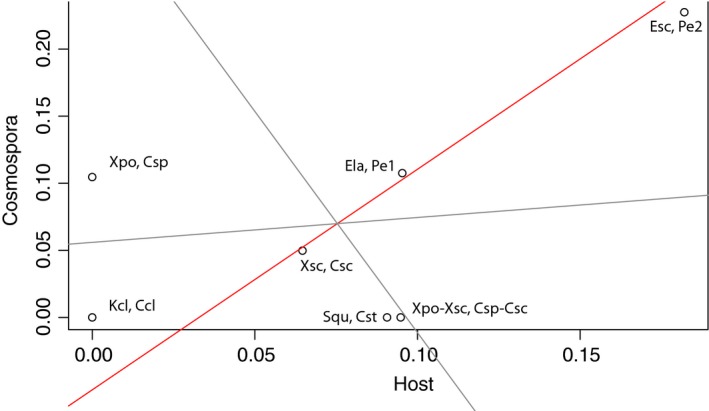
Plot of analogous host and parasite branches for terminal taxa of hosts and terminal taxa of *Cosmospora* based on fourfold‐degenerate sites. The slope of the line (Model II regression analysis) is 1.644 with a marginally significant *y*‐intercept (−0.054; *P* = 0.091). These results suggest that the rate of substitutions in *tub2* is roughly 1.5 times faster in *Cosmospora* compared to their hosts and that parasite divergence was slightly after the host divergence.

There were 58 and 62 fourfold‐degenerate sites in the *Cosmospora* and host matrices, respectively. Likelihood ratio tests failed to reject the null hypothesis of equal evolutionary rate throughout the tree (or molecular clock hypothesis; *P *>* *0.05). Model II regression analysis produced a slope value of 1.644 and a *y*‐intercept value of −0.054 (Fig. 7). The *y*‐intercept was marginally significant (*P *=* *0.091). These results suggest that the rate of substitutions in *tub2* is roughly 1.5 times faster in *Cosmospora* compared to their hosts and that parasite divergence was slightly after the host divergence.

## Discussion

Based on the results from this study, it can be inferred that pseudocospeciation (i.e., host switches followed by cospeciation) between *Cosmospora*‐like fungi and their xylariaceous hosts is a likely scenario. Distance‐based methods confirmed that the phylogenies of *Cosmospora* and the fungal host were more congruent than expected by chance (PACo, *P *=* *0.00001; ParaFit, *P *=* *0.005). The global congruence between host and parasite phylogenies has been interpreted as a result of cospeciation in many studies prior to this one (e.g., ants and plants, Itino et al. 2001; fungi and plants, Jackson 2004; penguins and their lice, Banks et al. 2006; mycoviruses and their fungal hosts, Göker et al. 2011; among others). However, in our study, not all individual host–parasite links were found to be coevolutionary (Fig. 4). Most host–parasite links considered coevolutionary included *Cosmospora* associated with *Kretzschmaria*,* Stilbohypoxylon*, and *Xylaria* hosts. These host genera represent recent evolutionary lineages of the Xylariaceae (Tang et al. 2009; Hsieh et al. 2010). Charleston and Robertson (2002) observed a similar global congruency of host–parasite cophylogenies and codivergences occurring at the tip of the host phylogeny. Given that there was a large difference in evolutionary rates between host and parasites, Charleston and Robertson (2002) determined that the observed evolutionary pattern could not be explained by cospeciation events alone, and suggested that this pattern was a result of host switches followed by cospeciation events.

Cospeciation is expected to have congruent phylogenies but also to have similar divergence times (Page 2003). Similar congruent topologies as seen in cospeciation could arise as a result of host switches followed by cospeciation events (or pseudocospeciation) but not have similar divergence times (Hafner and Nadler 1988; reviewed in Page 2003; De Vienne et al. 2007, 2013). Tree‐reconciliation‐based methods also supported the idea that the cophylogeny between *Cosmospora* and their fungal hosts could not be interpreted from strict cospeciation events (Figs. 5, 6). The reconciled trees contained five to six host‐switch events (Figs. 5, 6), which occurred early in the host phylogeny. Cospeciation events were more prevalent toward the tip of the host phylogeny.

We could not determine whether or not *Cosmospora* and the host have similar divergence times due to the lack of fossil records for fungi in general (Taylor and Berbee 2006). Calibration points are needed within the group of study to obtain more accurate estimates of divergence times. However, we were able to determine rates of evolution for *tub*2. The results indicated a marginally significant 1.6‐fold rate difference between *Cosmospora* and their hosts. Because the rates of evolution are not equal, cospeciation is unlikely to explain the apparent congruency between *Cosmospora* and the host phylogenies. The relatively high number of suspected host‐switch events in the reconciliation reconstructions also supports the idea that cospeciation is unlikely. These results suggest that pseudocospeciation represents a better hypothesis to explain congruent phylogenies with unequal rates of evolution.

Pseudocospeciation is often confused in the literature as cospeciation given the significant global congruency between host and parasite phylogenies, even though the parasites have been shown to diverge more recently than the host (Reed et al. 2007; Light and Hafner 2008). The lack of congruency in divergence times (or temporal congruency) between host and parasites should have refuted the hypothesis of cospeciation (Charleston and Robertson 2002; Sorenson et al. 2004; Huyse and Volckaert 2005). De Vienne et al. (2013) reviewed cospeciation literature and determined that only a small portion of the literature represented convincing cases of cospeciation. These cases involved symbionts that were transmitted vertically, which does not seem to be the case for *Cosmospora*. In contrast, Hafner and Nadler (1988) posited that pseudocospeciation resulted from host switches by the symbiont onto closely related hosts of the original host (horizontal transmission) followed by speciation on the new host. The resulting phylogenies of the host and the symbiont resemble the phylogenetic signature of cospeciation (i.e., cophylogenies) as result of the conserved host switching of the symbionts (Hafner and Nadler 1988; Charleston and Robertson 2002; Sorenson et al. 2004; Huyse and Volckaert 2005).

Host switching consists of a two‐step process (reviewed in Norton and Carpenter 1998). First, the acquisition of a new host by the parasite requires that the new host is found within the parasite's range and is related to the old host (i.e., phylogenetically similar; for example, Davies and Pedersen 2008), or has a similar ecological habitat to the old host (i.e., ecologically similar; i.e., Nikoh and Fukatsu 2000). Second, the parasite has to adapt to the new host in a way that diminishes gene flow between populations on the old host and populations on the new host. Lastly, the parasite on the new host will speciate as a result of limited gene flow over time. Host switching involves an initial decrease in host specificity during the colonization of a new host, and an increase in host specificity as speciation on the new host occurs (Norton and Carpenter 1998).

It may be difficult to explain how *Cosmospora* species are speciating and becoming host‐specific on xylariaceous fungi that live in sympatry. Congruence between host and pathogen phylogenies is not always evidence for widespread cospeciation because host shifts can give rise to congruent phylogenies if they occur mostly toward closely related hosts (De Vienne et al. 2007). This can be in part explained by the gene‐for‐gene coevolution hypothesis, a.k.a. matching gene coevolution (Thompson and Burdon 1992); or other variations, for example, inverse‐gene‐for‐gene hypothesis (Fenton et al. 2009). In host–parasite associations, infection occurs if host and parasite genes “recognize” (or not) each other. Thus, it is more likely that “virulence” gene(s) in *Cosmospora* mutate and infect a closely related host than it is to a distant host with phylogenetically distant resistance gene(s). In addition, because many of these xylariaceous hosts live in sympatry, there will be a higher chance for their spores to land on “suitable” hosts, because for symbionts, they must either cause infection or die. Both opportunities and selection for the utilization of a new host should therefore be frequent (Giraud et al. 2008).

In this study, we did not analyze demographic properties and its effects on divergence times because only *tub2* was available for comparison. It is important to consider that divergence patterns of a group of species will vary along their genomes due to polymorphism in the ancestors (Mailund et al. 2011). Therefore, different parts of the genome will have different histories because recombination has brought together the genome from different ancestors. To more accurately determine divergence time, it would be necessary to include other factors such as recombination rate, effective population size of the ancestral population, and the demographic effects and selection (Mailund et al. 2011; Werth et al. 2013).

This study represents a preliminary account of the evolutionary relationships between *Cosmospora*‐like fungi and their hosts, and further study of this group of fungi is likely to yield intriguing and complex results. Some species of *Cosmospora* sensu stricto are associated with basidiomycetes (Basidiomycota; Herrera et al. 2015; Gräfenhan et al. 2011), which could represent a putative interphylum host switch early in the evolution of *Cosmospora*. In other fungi, rapid speciation was observed after host switches, particularly those exploiting new adaptive zones (Zaffarano et al. 2008; Chaverri and Samuels 2013). Additionally, species of *Microcera* Desm. (Nectriaceae, Hypocreales, Ascomycota), a former group of fungi of *Cosmospora* sensu lato, are parasites of scale insects (Coccoidea, Hemiptera, Insecta; Gräfenhan et al. 2011) and lichens (Herrera & Chaverri, unpubl. data). This lineage of *Cosmospora*‐like fungi could represent a putative interkingdom host switch.

## Conflict of Interest

None declared.

## Supporting information


**Table S1.** Isolates of cosmospora‐like mycoparasites and corresponding accession numbers used in the phylogenetic analyses. **Table S2.** Isolates of the xylariaceous hosts and their accession numbers used in the phylogenetic analyses.
