# Supplementary material for: Pseudocospeciation of the mycoparasite Cosmospora with their fungal hosts
Source: Ecol Evol. 2016 Feb 9;6(5):1504–14. doi: 10.1002/ece3.1967 (PMC4775519; doi:10.1002/ece3.1967)
Supplement: Supplementary file 1 — Table S1. Isolates of cosmospora‐like mycoparasites and corresponding accession numbers used in the phylogenetic analyses. Table S2. Isolates of the xylariaceous hosts and their accession numbers used in the phylogenetic analyses. [file ECE3-6-1504-s001.docx]

Table S1. Isolates of cosmospora-like mycoparasites and corresponding accession numbers used in the phylogenetic analyses.

| **Species** | **ID code** | **Isolate No.** | **Herbarium No.** | **Fungal host** | **Geographic origin** | **GenBank Accession No.** | | | | |
| --- | --- | --- | --- | --- | --- | --- | --- | --- | --- | --- |
|  |  |  |  |  |  | **ITS** | **LSU** | **mcm7** | **rpb1** | **tub2** |
| *Cosmospora annulohypoxili* | Can | G.J.S. 96-186, CBS 137823 | BPI 744521 | *Annulohypoxylon* cf. *cohaerens* | USA | JN995635 | JN939816 | JN993308 | JQ031065 | KJ676285 |
| *Cosmospora arxii* | Car | G.J.S. 10-247, CBS 137811 | BPI 892899 | *Hypoxylon fragiforme* | USA | JN995629 | JN939824 | JN993326 | KC291869 | KC291908 |
| *Cosmospora clavi* | Ccl | G.J.S. 84-290, CBS 137826 | G.J.S. 1076 (NY) | *Kretzschmaria clavus* | Brazil | KJ676159 | KJ676196 | KJ676316 | KJ676233 | KJ676275 |
| *Cosmospora khandalensis* | Ckh | A.R. 4799, CBS 137833 | BPI 892901 | *Annulohypoxylon* sp. | Argentina | KJ676146 | KJ676183 | KJ676303 | KJ676220 | KJ676259 |
| *Cosmospora novaezelandica* | Cno | G.J.S. 83-197, CBS 124032 | PDD 46401 | *Annulohypoxylon bovei* | New Zealand | KC291732 | KC291777 | KJ676315 | KC291868 | KC291907 |
| *Cosmospora scruposae* | Csc | G.J.S. 86-331, CBS 137816 | GJS 4487 (NY) | *Xylaria scruposa* | French Guiana | JN995631 | JN939822 | JN993325 | JQ031070 | KJ676280 |
| *Cosmospora* sp. | Csp | KAS 3751 | - | *Xylaria* cf. *polymorpha* | Canada | KJ676173 | KJ676210 | – | KJ676247 | KJ676294 |
| *Cosmospora stilbohypoxili* | Cst | A.R. 4783, CBS 137834 | BPI 892897 | *Stilbohypoxylon quisquiliarum* | Argentina | KJ676144 | KJ676181 | KJ676301 | KJ676218 | KJ676257 |
| *Cosmospora ustulinae* | Cus | A.R. 4215, CBS 137835 | BPI 871089 | *Kretzschmaria deusta* | USA | JN995619 | JN939841 | JN993339 | JQ031088 | KJ676254 |
| *Dialonectria episphaeria* | Dep | G.J.S. 10-193, C.H. 10-01 | - | *Diatrype stigma* | USA | KC291744 | KC291771 | KC479773 | KC291892 | KC291932 |
| *Pseudocosmospora eutypae* | Pe1 | C.H. 11-01 | BPI 884164 | *Eutypa* sp. | France | KC291735 | KC291766 | KC291805 | KC291884 | KC291925 |
| *Pseudocosmospora eutypellae* | Pe2 | A.R. 4562 | BPI 884165 | *Eutypella* sp. | USA | KC291721 | KC291757 | KC291796 | KC291871 | KC291912 |
| *Pseudocosmospora joca* | Pjo | A.R. 4779 | BPI 884175 | *Biscogniauxia* sp. | Argentina | KC291746 | KC291762 | KC291801 | KC291887 | KC291924 |

Table S2. Isolates of the xylariaceous hosts and their accession numbers used in the phylogenetic analyses.

| **Species** | **ID code** | **Isolate/specimen no.** | **Geographic origin** | **Associated with:** | **GenBank Accession No.** | | | |
| --- | --- | --- | --- | --- | --- | --- | --- | --- |
|  |  |  |  |  | **BTUB** | **ACT** | **ITS** | **RPB2** |
| *Stilbohypoxylon quisquiliarum* | Squ | 172 (JDR) | French Guiana | *Cosmospora stilbohypoxili* | EF025605 | EF025590 | EF026119 | GQ853020 |
| *Kretzschmaria clavus* | Kcl | 114 (JDR) | French Guiana | *Cosmospora clavi* | EF025611 | EF025596 | EF026126 | GQ844789 |
| *Kretzschmaria deusta* | Kde | JF05154 | France | *Cosmospora ustulinae* | DQ840092 | - | - | - |
| *Annulohypoxylon bovei* | Abo | YMJ 90081914 | Taiwan | *Cosmospora novaezelandica* | AY951654 | AY951765 | EF026141 | - |
| *Hypoxylon fragiforme* | Hfr | YMJ 387 | France | *Cosmospora arxii* | AY951719 | AY951831 | JN979419 | - |
| *Biscogniauxia capnodes* | Bca | YMJ 138 | Taiwan | *Pseudocosmospora joca* | AY951675 | AY951787 | EF026131 | JX507779 |
| *Xylaria polymorpha* | Xpo | 1012 (JDR) | USA | *Cosmospora* sp. | GQ495954 | GQ452364 | GU322460 | GQ848343 |
| *Xylaria scruposa* | Xsc | CLL5025 | Martinique | *Cosmospora scruposae* | GQ495952 | GQ452362 | GU322458 | GQ848341 |
| *Annulohypoxylon cohaerens* | Aco | YMJ 310 | France | *Cosmospora annulohypoxili* | AY951655 | AY951766 | EF026140 | GQ844766 |
| *Hypoxylon "khandalensis"* | Asp | AR4799b | Argentina | *Cosmospora khandalensis* | - | - | XXXXXXX | - |
| *Diatrype stigma* | Dst | UCDDCash200 | USA | *Dialonectria episphaeria* | DQ007003 | - | DQ006945 | - |
| *Eutypa lata* | Ela | CBS 289.87 | France | *Pseudocosmospora eutypae* | DQ006973 | - | DQ006928 | - |
| *Eutypella scoparia* | Esc | DFMAL100 | USA? | *Pseudocosmospora eutypellae* | GQ294029 | - | GQ293962 | - |
